# Supplementary material for: MetaQTL: a package of new computational methods for the meta-analysis of QTL mapping experiments
Source: BMC Bioinformatics. 2007 Feb 8;8:49. doi: 10.1186/1471-2105-8-49 (PMC1808479; doi:10.1186/1471-2105-8-49)
Supplement: Additional File 7 — MetaQTL Package : jar file and tutorial. This Zip archive contains both the MetaQTL JAR file and the files of the tutorial. [file 1471-2105-8-49-S7.zip › org.inra.metaqtl/doc/org/metaqtl/bio/class-use/IBioOntology.html]

Uses of Interface org.metaqtl.bio.IBioOntology


|  |  |  |
| --- | --- | --- |
| **Overview** \| **Package** \| **Class** \| **Use** \| **Tree** \| **Deprecated** \| **Index** \| **Help** | |  |
| PREV   NEXT | **FRAMES**    **NO FRAMES**     **All Classes** |  |


---


## **Uses of Interface org.metaqtl.bio.IBioOntology**

| Packages that use IBioOntology | |
| --- | --- |
| **org.metaqtl** |  |
| **org.metaqtl.algo** |  |
| **org.metaqtl.bio.entity** |  |
| **org.metaqtl.main** |  |

Uses of IBioOntology in org.metaqtl

| Methods in org.metaqtl with parameters of type IBioOntology | |
| --- | --- |
| `void` | `MetaQtlData.doTraitOntologyClustering(IBioOntology ontology)`             According to the given trait ontology this methods clusterize the QTL in group in such a way that each group represents a trait term in the ontology. |

Uses of IBioOntology in org.metaqtl.algo

| Fields in org.metaqtl.algo declared as IBioOntology | |
| --- | --- |
| `protected  IBioOntology` | `ClustAlgorithm.ontology`             The optional trait ontology to use to group Qtl. |

Constructors in org.metaqtl.algo with parameters of type IBioOntology

`ClustAlgorithm(IBioGenome map,
IBioOntology ontology)`

`ClustAlgorithm(IBioGenome map,
java.lang.String name,
IBioOntology ontology)`

`QtlClustAlgorithm(IBioGenome map,
IBioOntology ontology)`

`QtlClustAlgorithm(IBioGenome map,
java.lang.String chrom,
IBioOntology ontology)`

`QtlTreeAlgorithm(IBioGenome map,
IBioOntology ontology)`

Uses of IBioOntology in org.metaqtl.bio.entity

| Classes in org.metaqtl.bio.entity that implement IBioOntology | |
| --- | --- |
| `class` | `Ontology` |

Uses of IBioOntology in org.metaqtl.main

| Methods in org.metaqtl.main that return IBioOntology | |
| --- | --- |
| `protected static IBioOntology` | `MetaMain.getOntology(java.lang.String ontoF, char format)` |

| Methods in org.metaqtl.main with parameters of type IBioOntology | |
| --- | --- |
| `static ChromGraph[]` | `MetaMain.getChromGraph(Chromosome[] chroms, boolean withQtl, int modeQtl, IBioOntology ontology, MetaQtlAnalysis treeAnalysis)` |
| `static QtlPartition` | `MetaMain.getQtlPartition(Chromosome chromosome, IBioOntology ontology)` |

---


|  |  |  |
| --- | --- | --- |
| **Overview** \| **Package** \| **Class** \| **Use** \| **Tree** \| **Deprecated** \| **Index** \| **Help** | |  |
| PREV   NEXT | **FRAMES**    **NO FRAMES**     **All Classes** |  |


---
